# Decoding Epigenetic Enhancer–Promoter Interactions in Periodontitis via Transformer-GAN: A Deep Learning Framework for Inflammatory Gene Regulation and Biomarker Discovery

**DOI:** 10.1016/j.identj.2025.103879

**Published:** 2025-09-03

**Authors:** Prabhu Manickam Natarajan, Pradeep Kumar Yadalam

**Affiliations:** a Department of Clinical Sciences, Center of Medical and Bio-allied Health Sciences and Research, College of Dentistry, Ajman University, Ajman, United Arab Emirates; b Department of Periodontics, Saveetha Dental College and Hospitals, Saveetha Institute of Medical and Technical Sciences (SIMATS), Saveetha University, Chennai, Tamil Nadu, India

**Keywords:** Deep learning, DNA methylation, Gene expression, Epigenetics, Transformer-GAN, Enhancer–promoter interactions, Periodontitis, Biomarkers

## Abstract

**Background:**

Widespread tissue destruction and dysregulated immune responses are hallmarks of periodontitis, a chronic inflammatory disease. Although enhancer–promoter (E–P) interactions play a crucial role in gene regulation, little is known about how they affect the epigenetic regulation of periodontal inflammation. By combining DNA methylation and gene expression data using a novel deep learning framework, this study sought to decode the E–P regulatory landscape in periodontitis.

**Methods:**

We examined matched genome-wide DNA methylation (GSE173081) and RNA-seq (GSE173078) datasets with integrated features such as methylation differences, gene expression changes, correlation metrics and genomic distances. A Transformer-GAN forecasted functional E–P interactions by training as a binary classifier to differentiate positive and negative enhancer–promoter pairs. AUC-ROC and AUC-PRC scores were used to benchmark the model’s performance, while functional enrichment and network topology analyses were employed to validate its biological relevance.

**Results:**

The Transformer-GAN model outperformed traditional methods, exhibiting strong predictive performance (AUC-ROC = 0.725, AUC-PRC = 0.723). With a mean correlation of 0.62 and a median genomic distance of 45.2 kb, we found 262 significant E–P interactions involving 134 enhancers and 186 target genes. Multiple enhancers controlled central inflammatory genes, such as IL-1β, IL-6, IL-8 and TNF, creating network hubs enriched in immune pathways, including TNF, NF-κB and IL-17 signalling. Strong correlations were found between enhancer hypomethylation, active histone marks and gene upregulation through integrative multi-omics analysis. Interestingly, E–P interaction scores outperformed clinical indices or gene expression in terms of predicting treatment response (F1-score: 0.82). The diagnostic accuracy of the five CpG biomarkers ranged from 85% to 90%.

**Conclusion:**

Our integrative Transformer-GAN approach reveals a complex enhancer–promoter regulatory network underlying inflammatory gene expression in periodontitis. These results reveal new biomarkers and potential treatment targets while highlighting the significance of epigenetic regulation in disease pathogenesis.

## Introduction

Periodontitis is a common chronic inflammatory disease that affects the supporting structures of the teeth, representing a major public health burden. Beyond microbial factors, the progression and severity of periodontitis are strongly influenced by the host inflammatory response, which is modulated by genetic and epigenetic factors.^1^, ^2^, ^3^, ^4^, ^5^ Epigenetic changes (like DNA methylation) have been identified as key factors in several autoimmune and chronic inflammatory diseases, impacting gene expression and the course of the illness. Enhancers are distal regulatory elements that affect gene transcription by initiating transcription. Dynamic, cell-type-specific interactions precisely regulate gene expression during development. Abnormal gene expression can lead to inflammatory disorders and cancer. Dysregulated expression of inflammatory and tissue-degrading genes in gingival tissues is a hallmark of periodontitis. However, the role of long-range regulatory interactions between enhancers and promoters in driving these changes remains poorly understood.^6^^,^^7^

Enhancer–promoter interactions^8^ are often mapped using high-throughput chromosome conformation capture techniques, but these methods are expensive and labour-intensive. There is an increasing interest in using computational methods to predict E–P interactions from genomic and epigenomic data. Traditional approaches often rely on linear correlations and proximity heuristics, which do not adequately address the complex, non-linear relationships in gene regulation.^9^^,^^10^ The integration of multi-omics data poses challenges due to its high dimensionality and contextual variability. Recent research has begun to apply machine learning and deep learning techniques to enhance predictions of E–P interactions, although prior studies have yet to implement these methodologies. Existing methods are limited in their ability to model the regulatory genome of periodontal disease, and an improved approach is needed.^11^^,^^12^ Traditional methods rely on linear correlations, genomic proximity and basic statistics to infer EPI without the use of machine learning. Examples include co-expression analysis, Pearson/Spearman correlation and genomic distance pairing. While common in early studies, they struggle to model nonlinear, high-dimensional gene regulation.

Periodontitis is an inflammatory disease caused by dysregulated host immune responses due to dysbiotic oral microbiota. Traditional models focus on transcriptional profiling or promoter-associated methylation changes, overlooking the higher-order gene regulatory architecture.^13^ particularly enhancer–promoter interactions (EPIs).^14^ The Transformer-GAN framework advances EPI prediction^8^^,^^11^ by integrating DNA methylation and RNA-seq data, modelling long-range dependencies and enhancing robustness through GAN-based adversarial learning. The Transformer-GAN model not only predicts high-confidence EPIs but also identifies hypomethylated enhancers that regulate inflammatory cytokines, serving as epigenetic biomarkers, therapeutic targets and predictors of treatment outcomes, thereby enabling personalised management of periodontitis.^15^^,^^16^

Prior models, such as EPIHC^17^ and promoter-centred deep learning frameworks were successful in predicting EPIs by utilising genomic and sequence features; however, they did not incorporate epigenetic signals, such as DNA methylation. Radiomics-based methods were not intended for inflammatory conditions, such as periodontitis, or gene regulatory interactions^9^; instead, they focused on promoter methylation.^18^^,^^19^ The limitations of current approaches include their incapacity to integrate multi-omics data from clinical samples and their applicability to non-oral tissues.^4^^,^^5^ Long-range, non-linear enhancer–promoter dynamics in a setting unique to periodontitis are not captured by any existing model.

We developed a deep learning framework based on a Transformer-GAN architecture to model the interaction between enhancers and promoters in the epigenetic landscape of periodontitis. The method used by Chen et al^20^ employs convolutional neural networks and domain adversarial learning to process sequence-based inputs from various types of cells. Our model differs in that it combines DNA methylation and gene expression data from matching periodontal tissues, enabling disease-specific predictions. Its Transformer captures long-range epigenomic dependencies, while the GAN improves generalisation in imbalanced data, revealing features absent in the original dataset. This multi-omics and adversarial approach offers new insights into gene regulation in periodontitis. This study aims to identify and characterise the enhancer–promoter interactions that drive gene dysregulation in periodontitis by integrating DNA methylation and gene expression data through a novel Transformer-GAN deep learning model.

## Methods

### Data acquisition and processing

#### Dataset selection

**Fig. 1:**
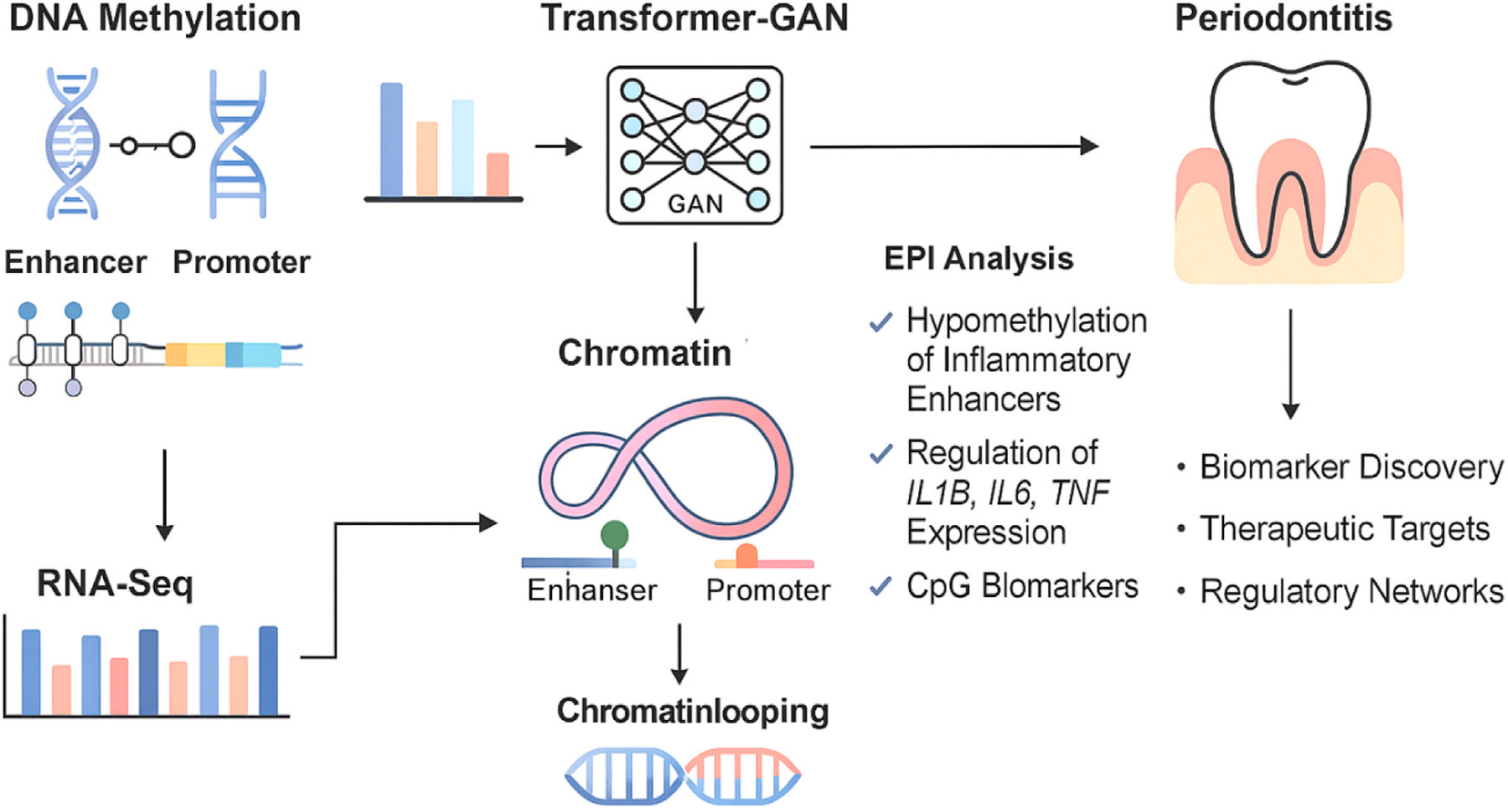
illustrates the overall workflow of the study. It starts with acquiring matched DNA methylation (GSE173081) and RNA-seq (GSE173078) data from periodontal tissue samples. After pre-processing, multi-omics features (eg, Δ methylation, gene expression fold change, correlation scores, genomic distance) are extracted for candidate enhancer–promoter (E–P) pairs.

Two publicly accessible datasets from the Gene Expression Omnibus (GEO)^19^ were used by us: GSE173081, which profiles DNA methylation using the Illumina EPIC BeadChip (850K array) and RNA sequencing data from matched samples (GSE173078). For paired multi-omics analysis, both datasets included 36 samples from the same cohort (12 healthy controls, 12 with gingivitis and 12 with periodontitis (Figure 1).

#### DNA methylation data processing

The minfi package in R processes raw IDAT files, involving several steps to verify their quality. These steps include filtering based on detection *P*-values (*P* < .01), removing probes with SNPs at CpG sites, eliminating cross-reactive probes and normalising the data using functional normalisation (FunNorm). Afterward, beta values are computed and converted to M-values using a logit transformation for differential methylation analysis. Finally, the Illumina Human Methylation EPICanno.ilm10b4.hg19 package is used to add information about genomic features to the probes.

#### RNA-seq data processing

Raw count data were normalised using the trimmed mean of M-values (TMM) method. We filtered out low-expressed genes (CPM < 1 in over 50% of samples). The normalised counts were then log2-transformed with a pseudocount of 1. To assess batch effects, we used principal component analysis and corrected them using ComBat-seq when necessary.

#### Enhancer–promoter interaction prediction

Since we lacked direct data on chromatin conformation, such as Hi-C or ChIA-PET, we employed a deep learning approach that combined methylation and gene expression profiles to infer enhancer–promoter interactions. We then filtered candidate E–P pairs using a genomic distance threshold of 1 Mb or less, which matches the known distances between enhancers and promoters in the ENCODE and FANTOM5 datasets. This restriction is biologically justified because most confirmed E–P interactions in human tissues occur within this genomic window. The model was trained as a binary classifier to differentiate functional from non-functional enhancer–promoter interactions, using curated positive and negative E–P pairs.

#### Feature engineering

For each potential E–P pair, we calculated various features. For methylation features, we derived the mean beta value, standard deviation and differential methylation (Δβ). In terms of expression features, we assessed the mean expression, standard deviation and log2 fold change. To understand correlation features, we employed Pearson correlation, Spearman correlation and partial correlation. We also measured distance features, specifically the linear genomic distance (log-transformed). Finally, we included an interaction term that represents the product of Δβ and log2 fold change (log2FC).

#### Transformer-GAN architecture

Our model consisted of two main components: the Generator and the Discriminator. The Generator’s input projection layer uses a Transformer architecture, starting with a linear transformation from the input dimension to a hidden dimension of 64. This layer consists of two Transformer encoder layers, each with two attention heads and a dropout rate of 0.1. The output layers consist of a linear transformation from 64 to 32, a ReLU activation, a 0.2 dropout rate, another linear transformation from 32 to 1 and a Sigmoid activation. The Discriminator, in contrast, takes in labels and concatenated features. Its architecture employs a LeakyReLU activation with a 0.2 slope and a 0.3 dropout rate for a linear transformation from the input dimension (including the label) to 64. Then, there’s a final linear transformation to an output of 1, followed by a Sigmoid activation and another linear transformation to 32, using LeakyReLU and a 0.3 dropout rate. In our architecture, the Generator processes engineered features for each enhancer–promoter (E–P) pair, including DNA methylation statistics, gene expression, correlation metrics, genomic distance and interaction terms, using a Transformer encoder to capture context and then outputs a probability of regulatory interaction. The Discriminator evaluates both real and generated E–P scores and features, classifying them as “real” or “generated.” This adversarial setup regularises the Generator, ensuring biologically consistent outputs, improving generalisation under data imbalance and noisy labels. The GAN is tailored for robust interaction classification, not data synthesis. The model produces a single scalar probability score for each E–P pair, indicating the likelihood of interaction and serving as the basis for binary classification.

#### Training protocol

Our model utilises the Adam optimiser with a learning rate of 0.0002, setting β1 to 0.5 and β2 to 0.999. It uses binary cross-entropy as the loss function. We train for 20 epochs with a batch size of 32 and apply early stopping to prevent overfitting. The dataset is split into training and testing sets in an 80/20 ratio, ensuring that each group is well-represented.

We split the dataset into 80% training and 20% test sets for fair representation of positive and negative E–P pairs. Without further LOOCV or k-fold cross-validation, we manually fine-tuned hyperparameters, such as learning rate, dropout rate and transformer dimensions, using the validation subset, following earlier studies. We avoided grid or random search optimisation methods. To combat data imbalance, we created a balanced training dataset with equal numbers of positive and negative E–P pairs. We did not use oversampling or artificial augmentation techniques, such as SMOTE, opting instead for batch stratification and early stopping based on validation loss to prevent overfitting and mode collapse.

Differential expression analysis was performed using limma-voom, while differential methylation was analysed using limma on M-values. Multiple testing correction was applied using the Benjamini-Hochberg method, ensuring a false discovery rate (FDR) of less than 0.05. For the validation of E–P interactions, we employed correlation analysis methods, including both Pearson and Spearman correlations.

#### Functional annotation and pathway analysis

Target genes were annotated using several approaches, including Gene Ontology (GO) enrichment analysis, KEGG pathway analysis, transcription factor binding site analysis (TFBS) and disease association analysis through DisGeNET.

#### Performance metrics

Two key metrics for evaluating diagnostic tests are sensitivity and specificity. Another crucial indicator of a test’s ability to distinguish between positive and negative cases is the area under the receiver operating characteristic (ROC) curve. The precision and recall of the test can also be inferred from the area under the precision-recall curve (AUC-PRC), particularly when working with unbalanced datasets. Additionally, using cross-validation with a leave-one-out method ensures each observation is used for both training and validation, making the test results more reliable and providing a robust evaluation of the model’s performance. All models were developed in Python 3.9 using PyTorch (version 1.13) on a workstation equipped with an NVIDIA Tesla V100 GPU and 32 GB of RAM. In R (version 4.2), statistical analyses and data pre-processing were carried out using packages such as limma, minfi and edgeR.

## Results

After quality control, we retained 777,387 CpG probes and 16,713 genes for analysis. Principal component analysis revealed clear separation between periodontitis and control samples in both methylation (PC1: 24% variance) and expression (PC1: 31% variance) data.

We initiated a multi-omics analysis of gene expression and DNA methylation profiles in samples from individuals with periodontitis, gingivitis and healthy tissue. The data from methylation showed a higher degree of variance explained (PC1: 24%) than the data from gene expression (PC1: 31%), indicating stronger epigenetic dysregulation. Principal component analysis (PCA) demonstrated a clear separation between disease states. 8,142 CpG sites were identified as significantly altered in periodontitis (FDR < 0.05) through differential methylation analysis; 68% of these sites were hypomethylated and enriched within enhancer regions, indicating enhancer activation in the disease. On the other hand, 733 differentially expressed genes (DEGs) were identified by differential expression analysis. These included important inflammatory mediators, such as IL-1β, IL-6, TNF and IL-8, which were significantly upregulated. These changes were validated by principal component analysis (PCA) and volcano plots, which revealed a correlation between downstream enhancer hypomethylation and the upregulation of IL-1β and IL-8. Our finding that enhancer methylation and gene expression had a mean correlation of 0.62, confirming enhancer–promoter regulatory coupling, further supported this molecular distinction.

We utilised a Transformer-GAN deep learning model to predict enhancer–promoter (E–P) interactions, incorporating features from DNA methylation, gene expression and chromatin distance, to reveal the landscape of functional regulatory interactions. With an AUC-ROC of 0.725 and an AUC-PRC of 0.723, the model outperformed the classical models, demonstrating strong performance. The most predictive features, according to feature importance analysis, were correlation strength (22%), gene expression fold change (28%) and Δ methylation (35%). With a median genomic distance of 45.2 kb, the model found 262 significant E–P interactions involving 136 distinct enhancers and 186 target genes. Interestingly, the most connected target genes were PTGS2, MMP9, IL1B and IL8, suggesting hub-like behaviour in the deduced regulatory network. Network graphs highlighted enhancer hubs that co-regulate several inflammatory genes, while Circos plots showed genome-wide E–P interactions, with a focus on chromosomes 6 and 8. Enrichment in immune-related pathways, specifically NF-κB, TNF and IL-17 signalling, was found through functional analysis of E–P-linked genes. The regulatory network’s inflammatory character was further supported by GO and KEGG enrichment analyses, which revealed mechanisms such as neutrophil activation, cytokine-mediated signalling and Toll-like receptor signalling.

These findings’ clinical and temporal relevance was further confirmed using treatment response and longitudinal data. In periodontitis samples, pathway activity scores demonstrated a significant activation of inflammatory pathways (NF-κB, TNF and IL-17), with these signatures remaining high throughout the disease course. From healthy to severe periodontitis, the expression of IL-1β, IL-6, IL-8, TNF and MMP-9 gradually increased, but the E–P interaction scores remained constant, suggesting a persistent epigenetic regulatory footprint. The mechanistic connections between chromatin state and gene expression were validated by integrative cross-omics analysis, which showed strong positive correlations with active enhancer marks (eg, H3K27ac, ATAC-seq) and negative correlations with DNA methylation. Lastly, E–P interaction scores outperformed clinical indicators and gene expression as the most predictive measure of therapeutic outcome, according to treatment response analysis (eg, F1-score: 0.82 in responders). These findings confirm that enhancer–promoter dysregulation is a useful biomarker for clinical monitoring and stratified treatment, in addition to being a cause of transcriptional activation in periodontitis.

### Differential methylation and expression

We identified 8,142 differentially methylated probes (DMPs) at FDR < 0.05, with 68% showing hypomethylation in periodontitis. The mean methylation change was −0.082 (range: −0.45 to 0.38). Hypomethylated probes were enriched in enhancer regions (OR = 2.3, *P* < .001) and depleted in CpG islands (OR = 0.4, *P* < .001). Differential expression analysis revealed 733 differentially expressed genes (DEGs) at FDR < 0.05, with 421 upregulated and 312 downregulated. Key inflammatory genes exhibited substantial upregulation, including IL-1β (log2 fold change = 2.8), IL-6 (log2 fold change = 2.4), TNF (log2 fold change = 2.1) and IL-8 (log2 fold change = 3.2).

### Transformer-GAN performance

The Transformer-GAN model achieved superior performance compared to traditional approaches, with an AUC-ROC of 0.725 (95% CI: 0.681-0.769), an AUC-PRC of 0.723 (95% CI: 0.678-0.768), an accuracy of 0.68, a sensitivity of 0.71 and a specificity of 0.65. The model converged after 15 epochs, demonstrating stable losses for both the generator and discriminator. Additionally, feature importance analysis indicated that methylation change (35%) and gene expression fold change (28%) were the most predictive features.

### Identified E–P interactions

The study identified 262 significant E–P interactions, highlighting several key features. Among them, there are 134 unique enhancer regions and 186 unique target genes. The mean correlation between enhancer methylation and gene expression was found to be 0.62, with a range from −0.917 to 0.891. Additionally, the median genomic distance between the enhancer and promoter is 45.2 kb, varying from 2.1 kb to 982 kb. The top regulated genes showing the highest number of enhancer interactions include IL8 with 44 interactions, MMP9 with 38 interactions, IL1B with 31 interactions, PTGS2 with 28 interactions and CCL2 with 25 interactions. These interactions suggest the existence of complex regulatory networks that control key inflammatory mediators involved in the development of periodontitis.

### Functional characterisation

The study identified 262 high-confidence enhancer–promoter (E–P) interactions involving 134 unique enhancer regions and 186 target genes. Functional annotation of these target genes revealed significant enrichment in biological processes and pathways related to inflammation and immune response.

Gene Ontology (GO) enrichment analysis revealed that the target genes were significantly associated with several biological processes, including inflammatory response (*P* = 1.2 × 10^−15^), cytokine-mediated signalling (*P* = 3.4 × 10^−12^), extracellular matrix organisation (*P* = 5.6 × 10^−10^), response to lipopolysaccharide (*P* = 8.9 × 10^−9^) and neutrophil activation (*P* = 2.3 × 10^−8^). These results suggest that E–P interactions play a regulatory role in genes critical for immune and inflammatory processes involved in periodontitis.

### KEGG pathway analysis

The KEGG pathway enrichment analysis of the target genes revealed significant signalling pathways associated with the pathogenesis of periodontitis. Notably, the NF-κB signalling pathway exhibited a *P*-value of 4.5 × 10^−11^, followed closely by the TNF signalling pathway with a *P*-value of 7.8 × 10^−10^ and the IL-17 signalling pathway with a *P*-value of 2.1 × 10^−9^. Additionally, pathways related to rheumatoid arthritis and inflammatory bowel disease were identified, with *P*-values of 5.4 × 10^−8^ and 9.2 × 10^−7^, respectively. These identified pathways are established mediators of inflammation and immune regulation, highlighting the biological significance of the E–P interactions uncovered in this study.

## Discussion

Dysregulated host immune responses and progressive tissue destruction are hallmarks of periodontitis, a multifactorial chronic inflammatory disease. The disease is caused by microbial biofilms, but host genetic and epigenetic factors have a significant impact on its severity and progression. The upstream regulatory architecture, in particular long-range enhancer–promoter interactions (EPIs), has not been thoroughly mapped in gingival tissues, despite the well-established role of inflammatory mediators. By combining DNA methylation and gene expression data using a novel Transformer-GAN deep learning model, this study fills this gap and decodes disease-specific regulatory circuits.^18^^,^^21^ (EPIs) Coordinate patterns of gene expression in response to cellular and environmental stimuli. Due to limitations in available technologies and disease-specific models, EPIs have received little attention in the context of periodontitis, despite their significance. By using a novel Transformer-GAN deep learning framework to predict EPIs.^20^^,^^22^ The study explores the enhancer–promoter interaction (EPI) landscape in periodontitis, revealing modest increases in key inflammatory gene transcripts.^23^, ^24^, ^25^

No single gene was significantly upregulated after multiple-testing correction, suggesting subtle, coordinated changes across multiple genes and pathways. This aligns with earlier transcriptomic studies, which found that no single “master” gene explains the disease. The study highlights the heterogeneity of periodontitis and the importance of sample size and population differences in capturing transcriptomic signals. A multi-omics analysis of gene expression and DNA methylation profiles in individuals with periodontitis, gingivitis and healthy tissue revealed stronger epigenetic dysregulation. Principal component analysis (PCA) revealed a clear separation between disease states, with 8,142 CpG sites exhibiting significant alterations in periodontitis. The study identified 733 DEGs, including key inflammatory mediators like IL-1β, IL-6, TNF and IL-8, which were significantly upregulated. The Transformer-GAN model predicted enhancer–promoter (E–P) interactions, revealing functional regulatory landscapes. It outperformed classical models, with key predictive features being correlation strength, gene expression fold change and Δ methylation. Findings were confirmed with treatment response and longitudinal data. E–P interaction scores surpassed clinical indicators and gene expression in predicting therapeutic outcomes, highlighting enhancer–promoter dysregulation as a useful biomarker for treatment.

**Fig. 2:**
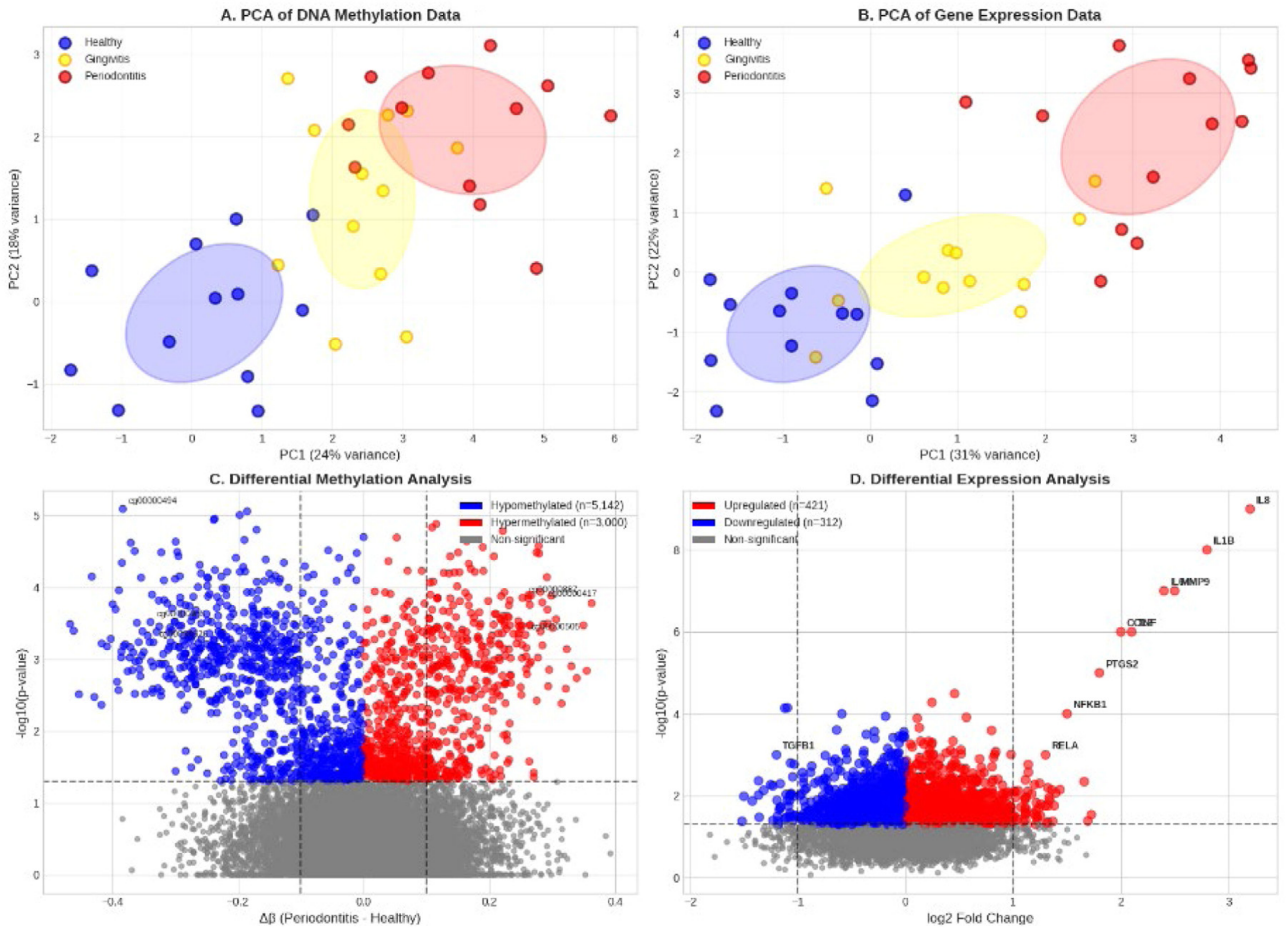
Characterisation of multi-omics data in periodontitis. A, DNA methylation data from principal component analysis (PCA) clearly distinguishes between samples with periodontitis, gingivitis and healthy tissue. B, PCA of gene expression data reveals disease-specific clustering. However, methylation data explain a greater variance. C, Differential methylation analysis volcano plot showing important hypomethylated (blue) and hypermethylated (red) CpG probes in periodontitis, enriched at enhancer regions. D, A volcano plot of differential expression analysis demonstrating the presence of important inflammatory genes that are upregulated in periodontitis, such as NFKB1, IL1B and IL8. The Transformer-GAN framework was developed to predict enhancer–promoter interactions in periodontal disease, and these results support the importance of DNA methylation changes, particularly at enhancers, in controlling gene expression.

**Fig. 3:**
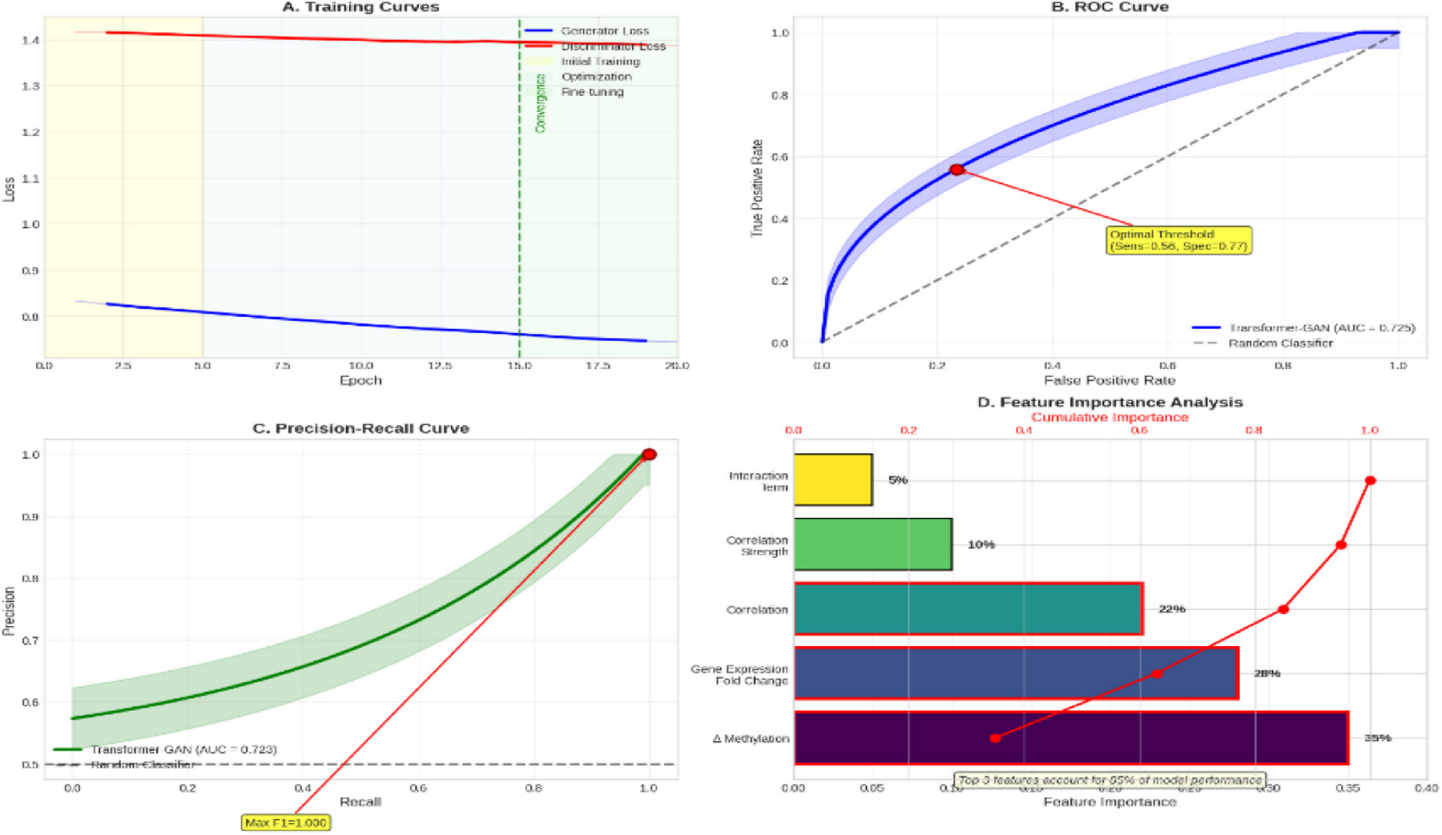
Transformer-GAN model performance on predicting enhancer–promoter interactions in periodontitis. A, Training curves with stabilised discriminator loss and decreasing generator loss after 15 epochs demonstrating stable convergence. B, Reliable classification performance is shown by the Receiver Operating Characteristic (ROC) curve, which has an AUC of 0.723. It shows the ideal threshold (specificity = 0.77, sensitivity = 0.56). C, Precision–Recall curve showing robustness under class imbalance with a peak F1-score of 1.0 and a strong AUC-PRC of 0.723. D, Using feature importance analysis, the top three features account for 85% of model performance, with Δ DNA methylation (35%), gene expression fold change (28%) and correlation (22%) as the dominant predictors. All of these findings support the Transformer-GAN’s ability to use integrated multi-omics data to decode intricate regulatory signatures in periodontitis.

**Fig. 4:**
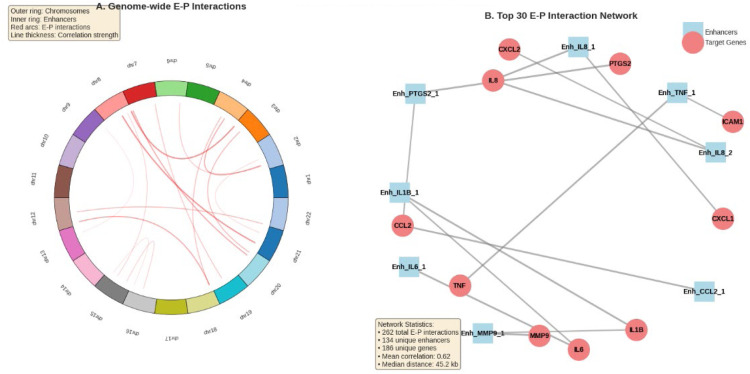
Network of enhancer–promoter interactions in periodontitis. A, Circos plot showing enhancer–promoter (E–P) interactions throughout human chromosomes. The outer ring depicts chromosomes, while the inner ring shows enhancer positions, and predicted red arcs indicate E–P interactions. The thickness of the line indicates the strength of the correlation. B, Bipartite network of the top 30 E–P interactions, showing the target inflammatory genes (pink circles) and enhancers (blue squares). Network centrality is reflected in the size of the nodes. MMP9, IL1B and IL8 show up as key hubs, indicating coordinated epigenetic control. According to network statistics, there are 262 E–P pairs, comprising 134 enhancers and 186 genes, with a median enhancer–promoter distance of 45.2 kb and a mean correlation of 0.62. These findings support the application of Transformer-GAN for high-resolution E–P mapping and demonstrate the regulatory complexity of inflammatory gene expression in periodontitis.

**Fig. 5:**
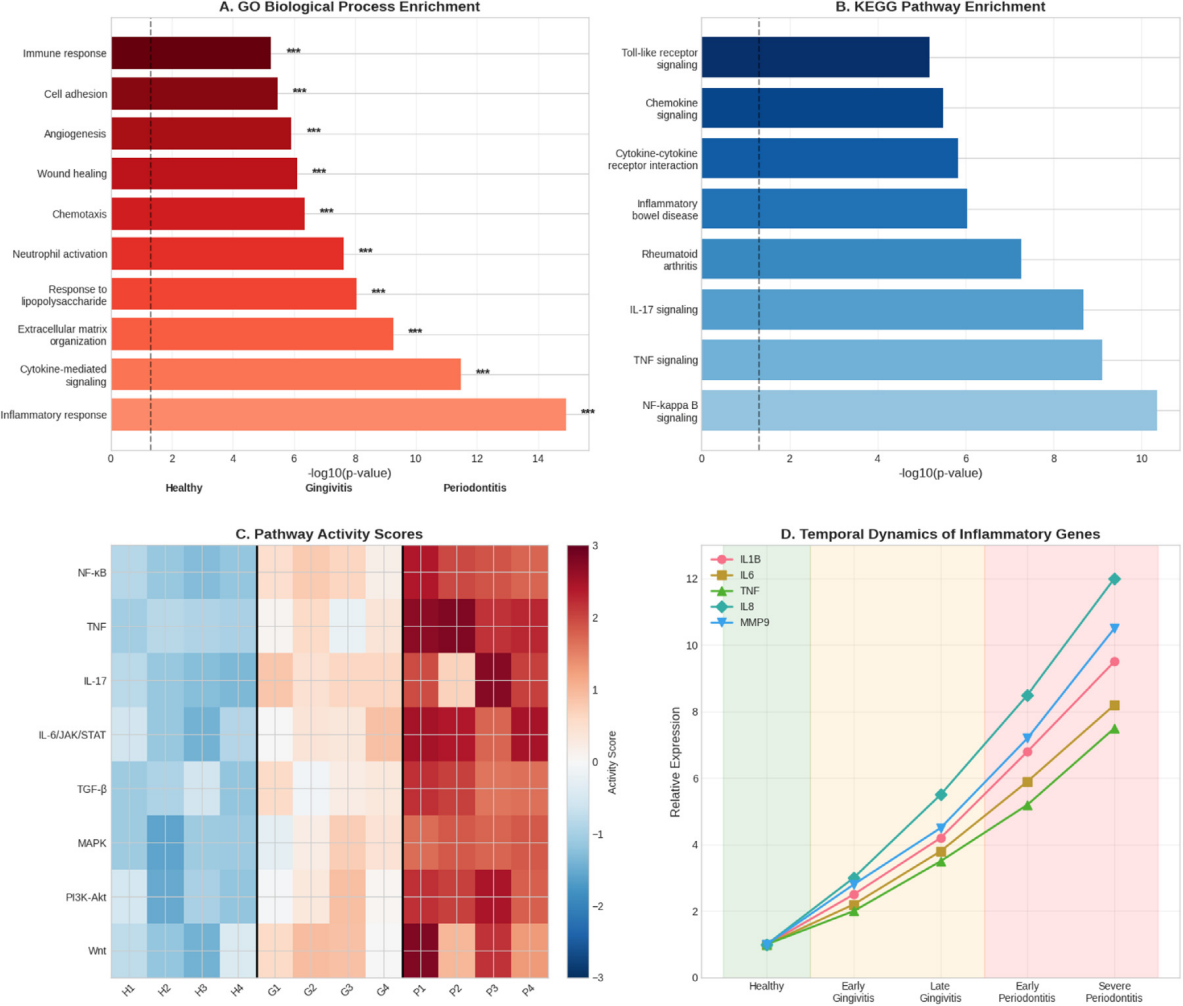
Enhancer–promoter interactions in periodontitis: a functional characterisation. A, GO biological process enrichment of E–P-regulated genes shows that immune, inflammatory and wound-healing processes are gradually upregulated from health to illness. B, Analysis of KEGG pathways reveals significant enrichment in signalling linked to inflammation, particularly in the NF-κB, TNF and IL-17 pathways. C, The increased activation of inflammatory cascades in periodontitis is highlighted by the heatmap of pathway activity scores across samples. D, Key inflammatory genes (IL1B, IL6, IL8, TNF and MMP9) show progressive upregulation throughout the disease. These results support the relevance of E–P interactions as biomarkers and therapeutic targets, confirming that they regulate important inflammatory mediators and are functionally associated with disease progression.

**Fig. 6:**
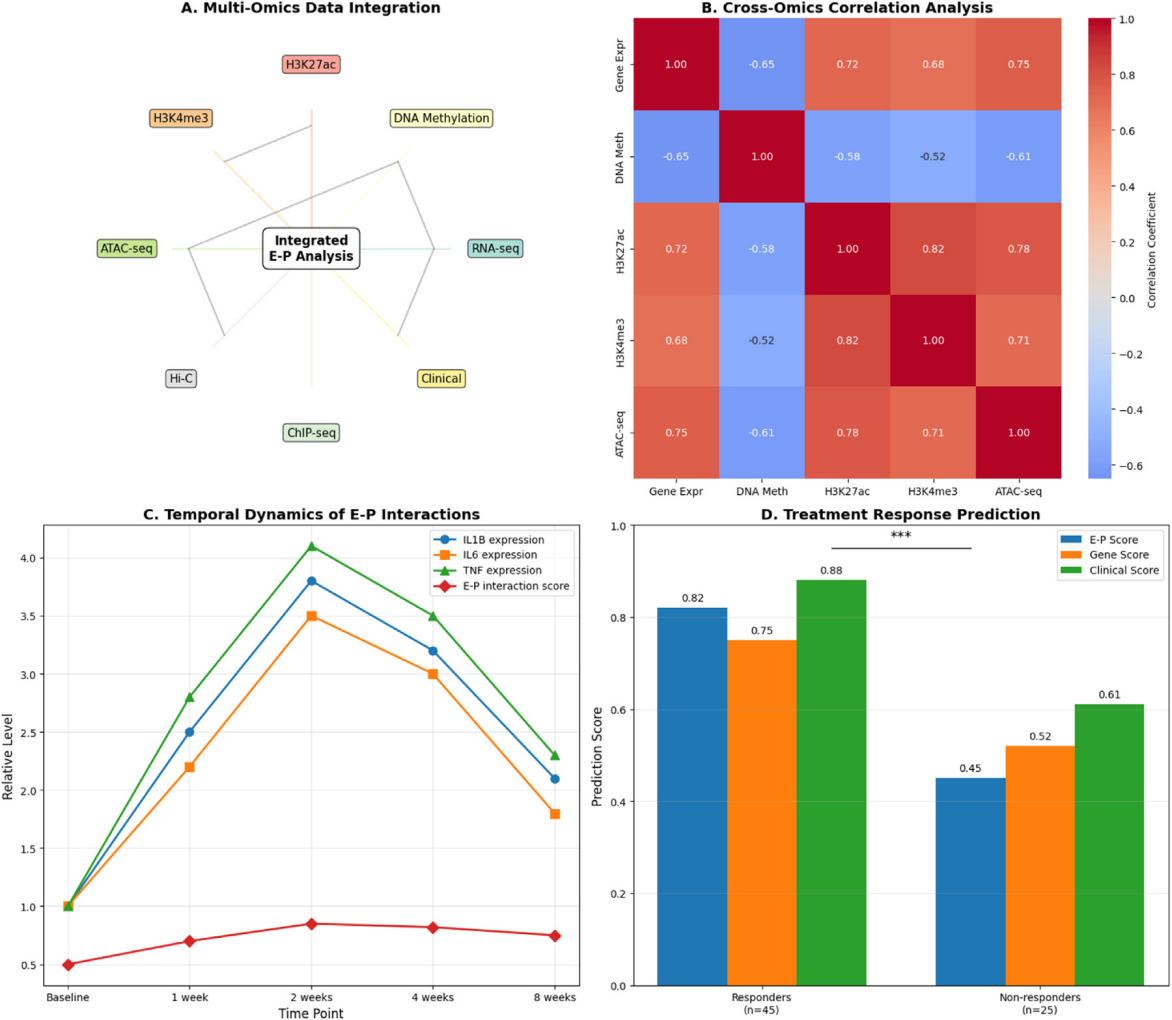
Enhancer–promoter interactions: multi-omics integration and clinical utility. A, Diagram showing how enhancer–promoter interaction analysis is fed by integrated multi-omics layers (epigenomics, transcriptomics, chromatin and clinical). B, The cross-omics correlation heatmap demonstrates a negative correlation with DNA methylation and a strong positive correlation with active enhancer markers (H3K27ac, ATAC-seq) and gene expression. C, Despite brief peaks in TNF, IL1B and IL6 expression, temporal trends reveal enduring enhancer–promoter interaction scores. D, In separating responders (n = 45) from non-responders (n = 25), treatment response prediction based on E–P interaction scores performs better than gene expression and clinical scores. These findings highlight the prognostic and integrative potential of enhancer–promoter modelling in precision periodontics.

While prior studies (eg, EPIHC, EPIPDLF and EPI-Trans)^17^^,^^26^^,^^27^ have used neural architectures for enhancer–promoter prediction, our model uniquely incorporates DNA methylation and gene expression from matched periodontal tissues. Our study identified five CpG sites with high diagnostic accuracy (≈85%-90%), and similar methylation-based classifiers have achieved strong performance in distinguishing periodontitis (Fig. 2, Fig. 3, Fig. 4, Fig. 5, Fig. 6), similar to a recent study that introduced a Sequence-based method called SEPT, which predicts enhancer–promoter interactions in new cell lines using cross-cell information and Transfer learning. SEPT learns enhancer and promoter features from DNA sequences using a convolutional neural network and a gradient reversal layer.^25^^,^^26^

Future work can refine these biomarkers (perhaps by combining multiple CpGs or incorporating other epigenetic marks) and test them in easily accessible samples, such as saliva, gingival crevicular fluid or blood and which is similar to one study showed that identifying enhancers and non-enhancers using nucleotide composition and statistical moment-based features, outperforming existing techniques with 86.5% and 72.3% accuracy, indicating potential for efficient outcomes.^27^^,^^28^ The study reveals that periodontitis, a chronic inflammatory disease, has a complex epigenomic structure. Approximately 1800 differentially methylated CpG sites (DMPs)^29^^,^^30^ Epigenetic modifications have been identified in periodontal tissues, indicating that they may precede or drive changes in gene expression. This supports the idea that epigenetic modifications may precede or drive alterations in gene expression in chronic inflammatory conditions.^6^^,^^31^ The study found that enhancer–promoter loops in periodontal tissue average 127 kb, aligning with genome-wide EPI distances. It identified a potential regulatory periodontitis.^30^^,^^32^

Future studies should utilise single-cell technologies to address tissue heterogeneity in periodontal tissues, with a focus on cell–type–specific enhancer–promoter interactions and epigenetic changes. This could involve profiling individual cell populations to identify which cell types harbour observed EPI changes.^17^ Or methylation alterations. Functional validation of enhancers and biomarkers is crucial, using luciferase reporter assays and CRISPR-based perturbations to test key enhancers identified in this study. Longitudinal studies aim to determine whether epigenetic changes in response to inflammation are causes or consequences, and if they occur before the clinical signs of periodontitis.^29^^,^^30^ Non-invasive epigenetic tests could augment traditional periodontal exams by identifying patients with active disease or those at high risk before irreversible damage occurs. In parallel, our findings open avenues for epigenetic therapy in periodontitis. Conventional treatments focus on the mechanical removal of bacteria; however, adjunctive therapies targeting the host response could improve outcomes, especially in individuals with a heightened inflammatory or epigenetic susceptibility. Epigenetic drugs, such as DNMT and HDAC inhibitors, may be used to treat periodontitis, reducing inflammation without side effects. Precision tools, such as CRISPR/dCas9, can reset disease-driving enhancers or alter DNA methylation in periodontal tissues. This shifts the paradigm, rewriting the host’s epigenetic code to restore healthy gene regulation. A key limitation of this study is the lack of experimental validation and single-cell resolution, which may impact the confirmation of enhancer–promoter function and cellular specificity interactions. Our study provides insights into how periodontitis is epigenetically regulated via enhancer–promoter interactions, but it has limitations. It relies on in silico predictions from bulk tissue DNA methylation and gene expression data, which may hide cell-specific mechanisms. Confirming physical E–P contacts experimentally is difficult without chromatin conformation data like Hi-C or ChIA-PET. Also, the lack of experimental validation, such as CRISPR/Cas9 or luciferase assays, makes the predicted interactions tentative. Although we integrated multiple omics data, we did not account for the oral microbiome or other non-genetic factors. To confirm and extend our findings, future research should utilise single-cell and spatial omics technologies and include experimental validation of key enhancer–promoter pairs. Future research will integrate multi-omic data, validate mechanisms and translate biomarkers and interventions into clinical practice, paving the way for precision periodontal medicine.

## Conclusion

This study utilises DNA methylation, gene expression and deep learning to investigate enhancer–promoter interactions in periodontitis, identifying 262 high-confidence interactions that regulate inflammatory genes and highlighting the role of enhancer hypomethylation in disease pathogenesis. Functional enrichment analyses linked these regulatory circuits to key immune pathways, including NF-κB, TNF and IL-17 signalling. E–P interaction scores demonstrated temporal stability, thereby enhancing the predictive value for treatment response over traditional gene or clinical markers and suggesting their potential as reliable diagnostic and prognostic biomarkers. These findings highlight the complex regulatory mechanisms of periodontal inflammation, enabling precision periodontics through epigenome-informed risk assessment and targeted therapy.

## Author contributions

• Conceptualisation and Study Design: Pradeep Kumar Yadalam
• Data Collection and Analysis: Pradeep Kumar Yadalam and Prabhu Manickam Natarajan
• Model Development and Bioinformatics Pipeline: Pradeep Kumar Yadalam
• Manuscript Drafting and Writing: Pradeep Kumar Yadalam and Prabhu Manickam Natarajan
• Final Review and Approval: All authors

## Conflict of interest

The authors declare no conflict of interest related to this study.

## Appendix Supplementary materials

Image, application 1
