# Corrigendum to ‘Decoding Epigenetic Enhancer–Promoter Interactions in Periodontitis via Transformer-GAN: A Deep Learning Framework for Inflammatory Gene Regulation and Biomarker Discovery’ [International Dental Journal Volume 75, Issue 6, December 2025, 103879]

**DOI:** 10.1016/j.identj.2025.103976

**Published:** 2025-11-06

**Authors:** Prabhu Manickam Natarajan, Pradeep Kumar Yadalam

**Affiliations:** a Department of Clinical Sciences, Center of Medical and Bio-allied Health Sciences and Research, College of Dentistry, Ajman University, Ajman, United Arab Emirates; b Department of Periodontics, Saveetha Dental College and Hospitals, Saveetha Institute of Medical and Technical Sciences (SIMATS), Saveetha University, Chennai, Tamil Nadu, India

The authors regret the following was not added to the **Acknowledgements** section of our published manuscript:

We would like to thank the Center of Medical and Bioallied Health Sciences and Research, Ajman University, Ajman, UAE.

The authors would like to apologise for any inconvenience caused.

